# Association between miRNA-145 and miRNA-155 expression in peripheral blood mononuclear cells of patients with multiple sclerosis: a case-control study

**DOI:** 10.1186/s12883-022-02909-6

**Published:** 2022-11-03

**Authors:** Sepide Ali Ashrafi, Milad Asadi, Dariush Shanehbandi, Saeed Sadigh Eteghad, Asra Fazlollahi, Seyed Aria Nejadghaderi, Sheida Shaafi

**Affiliations:** 1 Neurosciences Research Center (NSRC), Tabriz University of Medical Sciences, Tabriz, Iran; 2 Department of Basic Oncology, Health Institute of Ege University, Izmir, Turkey; 3 Immunology Research Center, Tabriz University of Medical Sciences, Tabriz, Iran; 4 Student Research Committee, Tabriz University of Medical Sciences, Tabriz, Iran; 5 Research Center for Integrative Medicine in Aging, Aging Research Institute, Tabriz University of Medical Sciences, Tabriz, Iran

**Keywords:** microRNA, miR-145, miR-155, Real-time PCR, Relapsing-remitting MS, Multiple sclerosis

## Abstract

**Introduction:**

MicroRNAs (miR or miRNA) are short regulatory RNAs, which modulate post-transcriptional gene expression. Dysregulation of these molecules contributes to pathogenicity of autoimmune disorders, such as multiple sclerosis (MS).

**Aims:**

This study was conducted to investigate changed expression pattern of miRNA-145 and miRNA-155 in MS.

**Methods:**

We collected blood samples of 75 patients with relapsing-remitting MS patients and 75 healthy controls. Ficoll-Hypaque density gradient method was used to isolate peripheral blood mononuclear cells. Also, total RNA was extracted and subjected to RT-PCR analysis. We used the Mann–Whitney U test to evaluate the differences in expression levels of target miRNAs between the groups.

**Results:**

We found that expression of miRNA-145 (P = 0.012) and miRNA-155 (P = 0.005) were partly reduced in patients with relapse-remitting MS in comparison with healthy controls. The miRNA-145 had an area under curve (AUC) of 0.621 (P = 0.01) and miRNA-155 levels had an AUC of 0.625 (P = 0.008).

**Conclusion:**

Decreased expression of miRNA-145 and miRNA-155 contributes to development of relapse-remitting MS, while further large scale observational studies and meta-analyses are required.

## Introduction

Multiple sclerosis (MS) is a multifactorial, immune-mediated disorder of the central nervous system (CNS) [1]. Its complex pathological processes lead to inflammatory demyelination and axonal damage of the CNS neurons, finally giving rise to neurological disability [1, 2]. The clinical course of MS can exhibit changing patterns over the time [3], but the usual course is characterized by a primary relapsing-remitting (RR) stage followed by the secondary progressive (SP) phase, and finally, the primary progressive (PP) phase, which is observed in only a small group of patients [4, 5, 6]. Similar to the clinical course and manifestations of MS, its response to therapeutic interventions, as well as the underlying pathogenicity mechanisms seems to be heterogeneous [7]. Biomarkers are considered as objective measures, which could be evaluated as indicators of regular biological processes, pathological processes or responses to therapeutic interventions [8]. Identification of valid biomarkers for MS bears a potential for a better diagnosis of MS, monitoring of the disease progress, and assessment of therapeutic responses [9].

MicroRNAs (miR or miRNA) are a class of short, widely-expressed, regulatory RNA molecules that modulate gene expression at the post-transcriptional stage [1]. Evidence suggests that these small molecules play a significant role in several cellular and physiological functions. Therefore, dysregulation of miRNA expression results in various pathological conditions, including autoimmunity and inflammation [10]. Recent studies have indicated that miRNAs circulate in a considerably stable form in human blood, supporting the strong potential for using them as promising diagnostic biomarkers [11, 12]. Investigations of miRNA expression profiles in RRMS, SPMS and PPMS patients indicate the differential expression of miRNAs in whole blood [13], peripheral blood mononuclear cells (PBMCs) [9, 10], B- and T-cell subsets [14–16], and active MS lesions [17]. These investigations have also proven that dysregulation of various miRNAs is closely related to development of MS [14–16].

Previous studies have suggested that the altered expression of miRNA-145 [18, 19] and miRNA-155 [20, 21, 22] contributes to the development of MS, and these molecules could serve as diagnostic biomarkers of the disease. However, the level of expression of the biomarkers in patients with MS in Iran have not been recently evaluated. So, in the present study, we compared the expression pattern of the mentioned candidate miRNAs in blood samples of patients with RRMS and healthy controls, in order to investigate their potential to be considered as diagnostic target for RRMS.

## Methods

### Study design and sample collection

In this case-control study, 75 patients with RRMS and 75 healthy controls were obtained from the Imam Reza Hospital, Tabriz University of Medical Sciences, Tabriz, Iran. Blood samples of the eligible participants were recruited between 2019 and 2020. The Expanded Disability Status Scale (EDSS) were calculated for the included patients with RRMS by an expert neurologist. The inclusion criteria were a diagnosis of MS according to the McDonald Diagnostic criteria [23] and being at least 18 years of age. The control participants should not have any history of neurodegenerative or mental disorders.

#### Written informed consent was obtained from all of the participants at the beginning of the study. The study protocol was approved by the ethics committee of Tabriz University of Medical Sciences (Ethics number

IR.TBZMED.REC.1400.433). All methods were performed in accordance with the national guidelines and regulations and the ethical standards of the Declaration of Helsinki 1964.

### RNA extraction

Following the preparation of blood samples from participants, PBMCs were isolated using a Ficoll-Hypaque density gradient. Following that, approximately 200 µl of PBMC samples were used for RNA extraction using Trizol reagent (Roche) according to the manufacturer’s instructions. Following the evaluation of the quality and quantity of extracted RNAs using the NanoDrop Spectrophotometer (NanoDrop ND-2000 C, Thermo Fisher Scientific, Massachusetts, USA), 1000 ng of the RNAs were reverse transcribed to complementary DNA (cDNA) using the microRNA First Strand cDNA Synthesis kit (EXIQON) as directed by the manufacturer.

### Genotyping

Real-time polymerase chain reaction (RT-PCR) assays were performed in the LightCycler® 96 Instrument using the BioFACTTM 2X Real-Time PCR Master Mix to evaluate the expression levels of target microRNAs. According to Schmittgen and Livak [24], the expression of target miRNAs was normalized to the U6 reference gene and evaluated using the 2 ^−∆CT^ approach. Each reaction was carried out three times in a total volume of 10 µl, with 5 µl of Master Mix, 2 µl of DEPC-treated water, 1 µl of Primer Mix, and 2 µl of cDNA. Table 1 shows the primer sequences. The reaction conditions were represented in Table 2.

**Table 1:** Target sequences for real-time polymerase chain reaction (RT-PCR) and primer sequences.

Micro-RNA	Stem loop	Forward primer	Reverse primer
miRNA-155	GTCGTATCCAGTGCAGGGTCCGAGGTATTCGCACTGGATACGAAACCCC	CGTGCTCATTTTAATGCTAATC	CCAGTGCAGGGTCCGAGGTA
miRNA-145	GTCGTATCCAGTGCAGGGTCCGAGGTATTCGCACTGGATACGAAGGGAT	CGTGCTCACGTCCAGTTTTC	CCAGTGCAGGGTCCGAGGTA
U6	GTCGTATCCAGTGCAGGGTCCGAGGTATTCGCACTGGATACGACAAAAATAT	GCTTCGGCAGCACATATACTAAAAT	CGCTTCACGAATTTGCGTGTCAT

**Table 2:** Conditions for the real-time polymerase chain reaction (RT-PCR).

Step	Tm	Time	Cycle namber
Pre-incubation	94c	600s	1
Denaturation	94c	10s	45
Annealing and Extension	60c	60s	
Melting			

### Statistical methods

The Mann–Whitney U test was used to assess the differences in target miRNA expression levels between the two groups. Furthermore, a receiver operating characteristic (ROC) analysis was performed to establish the discriminative capacity of miRNA levels. All data were expressed as mean and standard deviation (SD) of experiments, with a statistical significance level of P < 0.05. All of the graphics were created using the GraphPad Prism version 6.00 program (Graph Pad, San Diego, CA, USA).

## Results

The present study included a total of 150 participants, made up of 75 RRMS cases and 75 controls. There were 14 (19%) and 16 (21%) men among cases and controls, respectively. The mean age of participants was 34.5 (range: 22–75) in cases and 34.3 (range: 20–75) in controls. Among the cases, the age of onset of the disease was 27.7 ± 14.5 years old and time of diagnosis until sample collection was 17.3 ± 10.7 years. Moreover, the EDSS in patients were 4.12 ± 0.88.

**Fig. 1:**
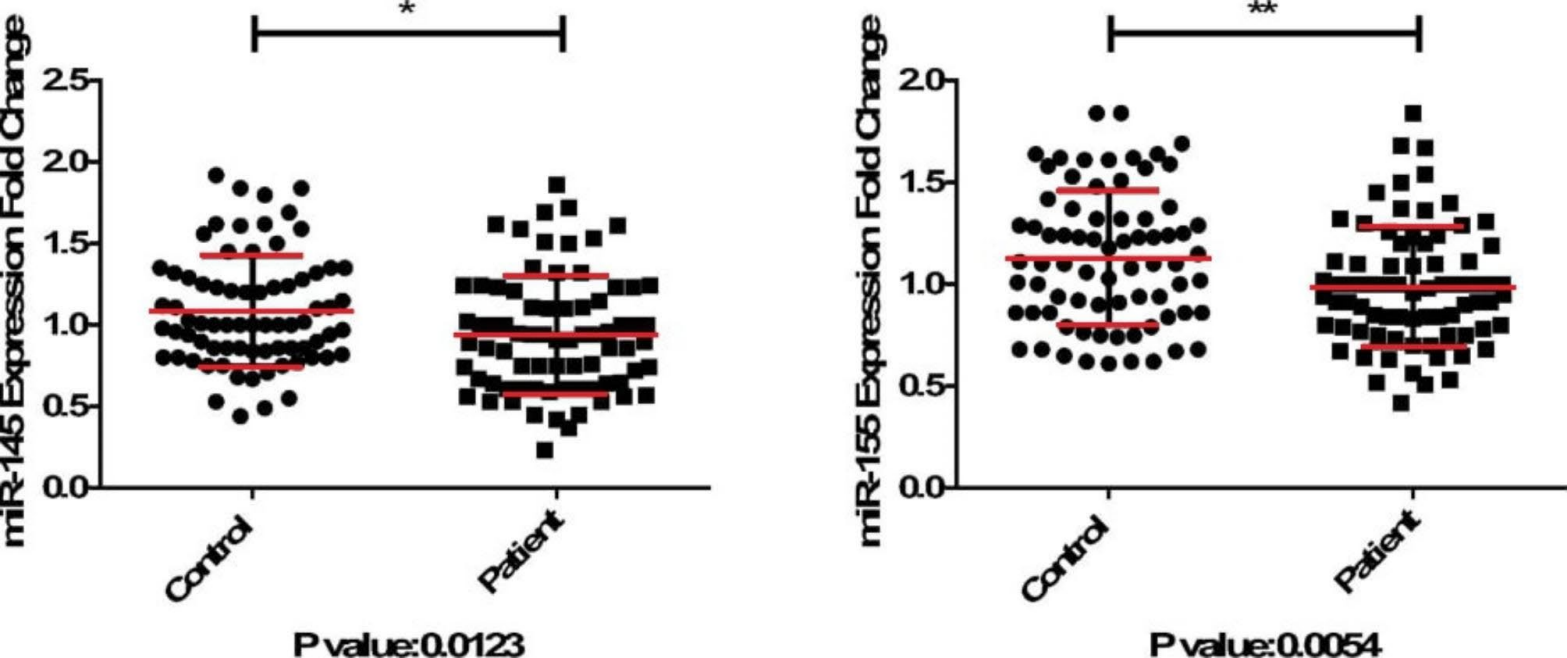
The relative expression of miRNA-145 and miRNA-155 in relapsing-remitting multiple sclerosis samples compared to healthy controls.

RT-PCR analysis demonstrated that expression of miRNA-145 and miRNA-155 were considerably lower in RRMS specimens in comparison with healthy controls (P = 0.012 and P = 0.005, respectively) (Fig. 1).

**Fig. 2:**
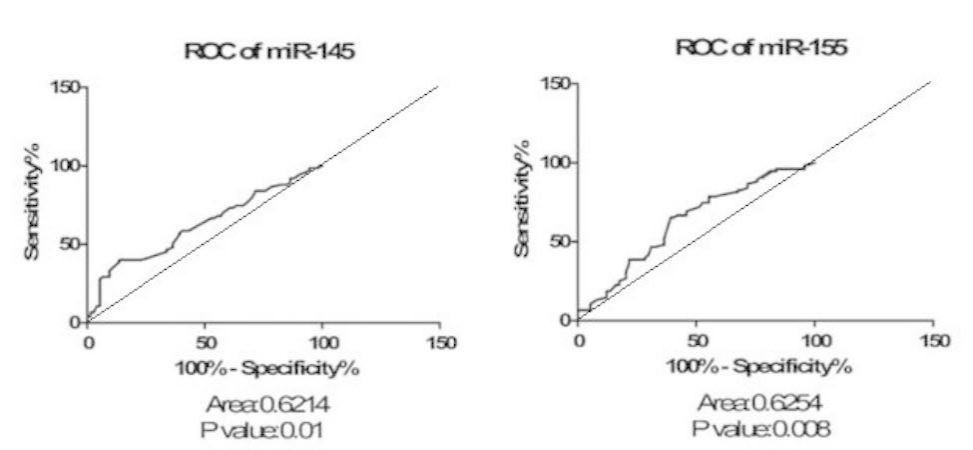
ROC curve of relapsing-remitting multiple sclerosis sample sets analyzed for relative expression level of miRNA-145 and miRNA-155. A diagonal line cutting the coordinates 0,0 and 100,100 with an AUC of 0.5 has been drawn. The difference between this line and the ROC curve of the proposed biomarker is the graphical representation of the quality of the diagnostic biomarker and could be helpful to easily evaluate the given ROC analysis.

ROC curve analysis was performed to assess the potential diagnostic value of the studied miRNAs and a notable level in separating RRMS and controls showed that miRNA-145 had an area under the curve (AUC) of 0.621 (P = 0.01) and miRNA-155 levels had an AUC of 0.625 (P = 0.008), suggesting that both of these miRNAs could be applied as clinical biomarkers for evaluation of MS (Fig. 2).

## Discussion

Findings of the present study showed that there was a significant difference between patients with RRMS and controls in terms of miRNA-145 and miRNA-155 expression which was in favor of the control group.

The miRNA-155 is an inflammation regulator and plays roles in MS development by different mechanisms [22]. In this regard, it can lead to blood-brain barrier disruption, promote demyelination processes and neuropathic pain which can be associated with neuropsychiatric complications like depression and anxiety in patients with MS [22]. In the article by Aljawadi and colleagues miRNA-155 was significantly higher expressed in peripheral blood leukocytes of 25 untreated MS patients compared to 25 healthy controls (p < 0.05) [25]. A previous study conducted by Paraboschit et al. on 10 patients with RRMS and 10 age- and sex-matched controls showed a close to significant results of higher expression of miRNA-155 in cases than controls (p = 0.053) [26]. Despite the findings of the study, we found that miRNA-155 was significantly higher presented in controls, not patients with MS (p = 0.005). It should be noted that the abovementioned study revealed a close to significant level and also different populations and measurements can lead to the variations between the findings. Moreover, the higher level of significance or lower p-values can be adjusted by larger sample size in our study.

The miRNA-145 is an miRNA which its down- or up-regulations are associated with different cancers (e.g. breast and gastric cancers), non-cancerous disorders (e.g. anemia, asthma, biliary atresia, diabetic nephropathy, and hepatopulmonary syndrome), and MS [18, 27]. A case-control study on 37 patients with MS, including 18 with RRMS and 19 with secondary progressive MS and 23 healthy controls showed a significantly higher expression of miRNA-145 in MS patients than controls (0.028 vs. 0.013; p = 0.028) [19]. The study also found an AUC of 0.67 (p = 0.028) which is close to our findings (AUC = 0.62), and represents that it could be used for discrimination of MS from healthy subjects [19]. Another study conducted by Keller and colleagues on 20 patients with RRMS and 19 controls revealed a significantly upregulated of has-miRNA-145 in MS patients (p < 0.0001) [9]. Moreover, it was the most remarkable dysregulated miRNA out of 866 assessed miRNAs which had an AUC value of 0.96 [9]. The higher AUC values of the study compared with our study could be due to different methodologies and objectives of the two study. The study by Aljawadi et al. also evaluated the expression levels of miRNA-145 among Iraqi patients with MS and revealed no significant difference between cases and controls in terms of miRNA-145 expression (p > 0.05) [25]. Another study also showed upregulation of miRNA-145 and highlighted the potential roles of this miRNA as diagnostic biomarker in PBMCs of MS patients (AUC value = 0.785; p = 0.0004) [1]. On the other hand, we found a remarkable higher expression of the miRNA-145 in controls. The differences in sample sizes and inclusion criteria for included patients could lead to the variations in the results.

It is one of the first and most recent studies which evaluated the expression of the two circulating miRNAs involved in MS development among the Iranian populations. Nevertheless, we acknowledge that our study has several limitations. Firstly, some clinical data such as administered treatments for the cases were not reported. Secondly, the findings could not be representative for other populations and regions since we collected data from residents of one of the cities in Iran. Therefore, sampling of other races or ethnicities might lead to different results. Thirdly, we used plasma serum of participants as a non-invasive method to measure plasma serum levels of participants instead of measuring the levels of miRNAs expression in different cell populations. Fourthly, the effects of genetic polymorphisms in the development of MS and its differences between patients with MS and healthy controls have not been evaluated, as a cohort on Egyptian patients with MS revealed that TT genotype and T allele in miRNA-155 were associated with elevated risk of MS [21]. Fifthly, we only included patients with RRMS, while other subtypes of MS were not included in the present study, so we cannot evaluate the roles of miRNA on distinguishing different MS subtypes. In this regard, the study by Gandhi et al. represented that miRNA-145-5p is significantly high regulated in RRMS than secondary progressive MS (p = 0.01) [28].

## Conclusion

The presented data revealed that miRNA-145 and miRNA-155 expression is partly reduced in RRMS. Since our study only includes patients with RRMS and healthy controls, further investigations and large scale observational studies are needed to evaluate the expression of mentioned miRNAs in other MS subtypes. Also, the underlying mechanism of these miRNAs in MS pathogenesis should be explained in future studies.

## Data Availability

The data that support the findings of this study are available on request from the corresponding author. The data are not publicly available due to privacy or ethical restrictions.

## List of abbreviations

MS: Multiple sclerosis.
CNS: central nervous system.
RR: relapsing-remitting.
SP: secondary progressive.
PP: primary progressive.
miR: MicroRNA.
mi-RNA: MicroRNA.
PBMC: peripheral blood mononuclear cell.
EDSS: Expanded Disability Status Scale.
cDNA: complementary DNA.
RT-PCR: Real-time polymerase chain reaction.
ROC: receiver operating characteristic.
SD: standard deviation.
AUC: area under the curve.
